# A 5-year comparison of marginal bone level following immediate loading of single-tooth implants placed in healed alveolar ridges and extraction sockets in the maxilla

**DOI:** 10.3389/fphys.2014.00029

**Published:** 2014-01-31

**Authors:** Antoine N. Berberi, Joseph M. Sabbagh, Moustafa N. Aboushelib, Ziad F. Noujeim, Ziad A. Salameh

**Affiliations:** ^1^Department of Oral and Maxillofacial Surgery, School of Dentistry, Lebanese UniversityBeirut, Lebanon; ^2^Department of Restorative Dentistry, School of Dentistry, Lebanese UniversityBeirut, Lebanon; ^3^Department of Research, School of Dentistry, Lebanese UniversityBeirut, Lebanon

**Keywords:** marginal bone level, peri-apical radiographs, dental implant, immediate loading, maxillae, healed sockets, fresh extraction sockets, single-tooth

## Abstract

**Purpose:** The aim of present investigation was to evaluate marginal bone level after 5-year follow-up of implants placed in healed ridges and fresh extraction sockets in maxilla with immediate loading protocol.

**Materials and Methods:** Thirty-six patients in need of a single-tooth replacement in the anterior maxilla received 42 Astra Tech implants (Astra Tech Implant system™, Dentsply Implants, Mölndal, Sweden). Implants were placed either in healed ridges (group I) or immediately into fresh extraction sockets (group II). Implants were restored and placed into functional loading immediately by using a prefabricated abutment. Marginal bone level relative to the implant reference point was recorded at implant placement, crown cementation, 12, 36, and 60 months following loading using intra-oral radiographs. Measurements were made on the mesial and distal sides of each implant.

**Results:** Overall, two implants were lost from the group II, before final crown cementation: they were excluded from the study. The mean change in marginal bone loss (MBL) after implant placement was 0.26 ± 0.161 mm for 1 year, and 0.26 ± 0.171 mm for 3 years, and 0.21 ± 0.185 mm for 5 years in extraction sockets and was 0.26 ± 0.176 mm for 1 year and 0.21 ± 0.175 mm for 3 years, and 0.19 ± 0.172 mm for 5 years in healed ridges group. Significant reduction of marginal bone was more pronounced in implants inserted in healed ridges (*P* < 0.041) compared to fresh surgical extraction sockets (*P* < 0.540). Significant MBL was observed on the mesial side of the implant after cementation of the provisional (*P* < 0.007) and after 12 months (*P* < 0.034) compared to the distal side which remained stable for 3 and 5 years observation period.

**Conclusions:** Within the limitations of this study, responses of local bone to immediately loaded implants placed either in extraction sockets or healed ridges were similar. Functional loading technique by using prefabricated abutment placed during the surgery time seems to maintain marginal bone around implant in both healed and fresh extraction sites.

## Introduction

Several criteria were proposed for evaluation of dental implants success. Commonly used criterion was suggested by Albrektsson et al. (1986), which was further reviewed in 1993 (Albrektsson and Zarb, 1993). According to Albrektsson and Isidor (1993), a successful implant should sustain less than 1.5 mm of bone loss during the first year in function, and less than 0.2 mm annually thereafter. In 1999, Wennström and Palmer (1999) suggested a modification of the radiological criteria regarding bone loss. They suggested that a maximal bone loss of 2 mm could be accepted over a 5-year period after loading of the prosthetic restoration.

Marginal bone loss (MBL) could be influenced by several surgical and prosthetic factors such as surgical trauma, occlusal overload, peri-implantitis, micro gap, biologic width, and implant macroscopic and microscopic characteristics at neck region in contact with bone, implant abutment interface design, flapless or flapped procedures, immediate insertion of implants in fresh extraction sockets, time of fixation of super structure, and time of loading (Quian et al., 2012). An immediate implant placement is defined as an implant placed in a fresh extraction socket immediately following tooth extraction (Schropp and Isidor, 2008). The placement of implants in fresh extraction sockets allows placement of the implant during the same visit when the tooth is extracted, and this reduces morbidity; decreases treatment time by reducing surgical procedures and may enhance esthetics. Furthermore, placement of an implant immediately after tooth extraction may help to maintain crestal bone and leads to ideal implant positioning from a prosthetic point of view (Scarano et al., 2000; Vanden Bogaerde et al., 2005; Crespi et al., 2007; Degidi et al., 2007). Immediate loading implies that the prosthesis is attached to the implant on the same day (Henry and Liddelow, 2008). Esposito and co-workers published a meta-analysis comparing success rates between immediately, early, and conventionally loaded implants. They found no statistically significant differences between the times of loading as long as a high degree of primary implant stability can be achieved (Esposito et al., 2007).

Immediate loading protocols have shown acceptable prosthodontics results and patient satisfaction outcomes in healed ridges and fresh extraction sockets (Kan et al., 2003; Norton, 2004; Cornelini et al., 2005; Barone et al., 2006; Lindeboom et al., 2006; Canullo and Rasperini, 2007; Hall et al., 2007; Crespi et al., 2008; De Rouck et al., 2008, 2009).

Following implant, abutment and crown placement, peri-implant soft tissue changes include papilla as well as midfacial recession of about 0.5–1.0 mm (Henriksson and Jemt, 2004; Cardaropoli et al., 2006; Cooper et al., 2007, 2010; De Bruyn et al., 2013). Recession has been described to occur for conventionally installed implants (Cardaropoli et al., 2006; Raes et al., 2011) as well as immediately installed implants (De Rouck et al., 2008; Raes et al., 2011). The non-removal of abutments placed during the surgery results in a statistically significant reduction of the horizontal bone around the immediately restored implants (Berglundh et al., 2005). The microgap between implant-abutment and disruption of the soft tissue that occurs each time the two are disconnected and reconnected, are thought to influence bone resorption around implant neck. Some amount of bone resorption occurs during the first year of loading (Hermann et al., 2001).

The aim of this study was to compare the MBL around single titanium implants placed in healed or fresh extraction sites in the maxilla and immediately loaded over a period of 5 years. The tested hypothesis was that more MBL would be observed in immediately loaded implants inserted in fresh extraction sockets compared to implants inserted in healed sockets.

## Materials and methods

This study was conducted in coherence with the Helsinki agreement for research on Human subjects (Carlson et al., 2004).

Inclusion criteria:

1. Age between 20 and 60 years,
2. Single dental implant required in the anterior maxilla,
3. Adequate bone volume to receive an implant
4. Natural teeth present both mesial and distal to the implant site,
5. More than 5 mm of bone height apical to the extraction socket,
6. Implants with at least 32 N/cm of initial stability, for the immediate loading protocol.

Exclusion criteria:

1. Previous bone grafting or bone regeneration in the area of implant placement,
2. Insufficient bone volume, needing bone regeneration or bone augmentation before implant placement,
3. Chronic peri-apical lesions of endodontic origin in the implant site,
4. Uncontrolled periodontal disease,
5. Medical condition or medication that might compromise healing or osseointegration,
6. Systemic diseases contradicting oral surgery,
7. Smoking more than 20 cigarettes per day for the last 3 years.
8. Unrealistic expectations in term of aesthetic results.

Following these criteria, 36 patients in need of single implant placement in anterior maxillae (21 males, 15 females, mean age of 31 years) were enrolled in this study. Selected cases included 21 central incisors, 13 lateral incisors, 1 canine, and 7 premolars, all handled by the same oral surgeon. Forty-two implants were placed in the anterior maxilla, 20 implants (10 central incisors, 7 lateral incisors, and 3 premolars) were placed in existent healed sockets (group I) and 22 implants (11 central incisors, 6 lateral incisors, 1 canine, and 4 premolars) were placed in fresh extraction sockets after extraction of the diseased teeth (group II). The implant dimensions were chosen to ensure optimal initial stability and dimensions ranged between 3.5/4.0 mm for lateral incisors and 4.5/5.0 for the rest in width and 11–15 mm in length.

### Implant placement

Implant placement was performed under local analgesia (2% Articaine 1; 100,000 adrenaline, 3 M Espe, Seefeld, Germany) and antibiotic treatment (2 g Amoxicillin or 600 mg Dalacin C for penicillin-allergic patients) then 1 g/300 mg b.i.d. for 5 days. Analgesic medication (Ibuprofen 600 mg, Abbott Healthcare Products Limited, UK) was also prescribed 1 h prior to the surgery. Following reflection of muco-periostal flap, for healed sockets (group I), titanium implants (Astra Tech Implant system, Dentsply Implants, Mölndal, Sweden) were inserted in drilling site using successive drill sizes and countersink to a level approximately 2 to 3 mm apical to the cement-enamel junction of the adjacent teeth. For extraction sockets (group II), osteotomies were directed through palatal aspect of the socket for a better primary stability and to keep the buccal plate intact. Any gaps between implants and socket were filled with patient's bone chips collected during the drilling procedure.

### Immediate temporization

Immediate temporizations were enabled by using the Ti Design™ or Zir Design™ abutments (Astra Tech Implant system™, Dentsply Implants, Mölndal, Sweden), which provided a restorative margin of approximately 1.5 mm below mucosal margin. Abutments were tightened at 10 N/cm with a torque controller. Temporary crowns (ProTemp Garant III, 3M ESPE America, Norristown, PA) were adapted directly in the mouth, than highly polished and cemented using a temporary cement (TempBond NE™, Kerr Hawe, S.A. CH). Excess cement was removed and provisional crowns were placed with demonstrable contacts in maximum intercuspal position with no eccentric or lateral contacts. Flaps were adjusted and secured around cemented restorations by means of single sutures (Vicryl^®^4/0, Johnson & Johnson Medical Limited, UK).

Oral hygiene instructions were given to the patients and include mouth rinses (0.12% digluconate chlorhexidine), 3 min each 3 h and after each meal, for 2 weeks period).

Two implants were lost before the final restoration procedure, due to infection problem from the group II, one central and one premolar. They were excluded from the study.

### Final restorations

Eight weeks after implant placement, the provisional crown was removed and the abutment was tightened with a torque controller (according to the manufacturers' recommendations). An impression with polyvinyl siloxane was taken and a full ceramic crown (Empress 2^®^ Ivoclar Vivadent AG, Liechtenstein) was fabricated using conventional prosthodontic procedures. The final crown was cemented with self-adhesive cement (Multilink, Ivoclar Vivadent AG, Liechtenstein).

### Radiological assessment protocol

**Figure 1:**
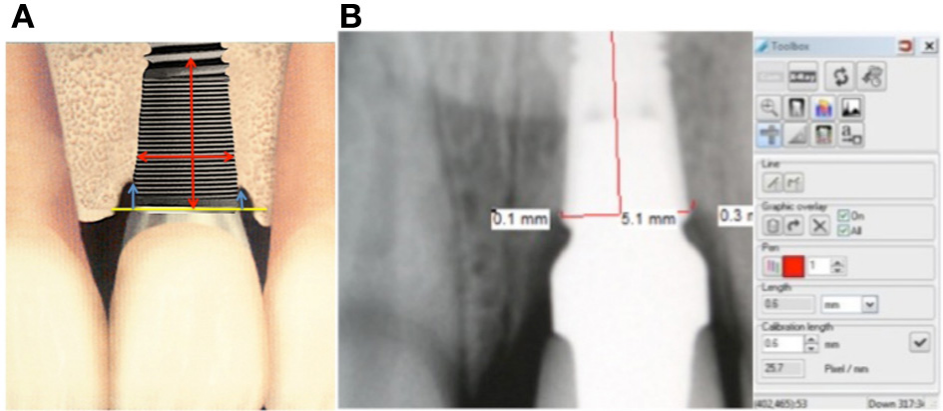
**Measurement technique. (A)** Mesial and distal marginal bone loss calculated as vertical distance between crestal bone level and implant neck; **(B)** Software calculation of the MBL.

Standardized periapical radiographs were obtained using a long-cone paralleling technique, with the central beam perpendicular to the alveolar crest (XCP holder Rinn™, Dentsply International, York, PA, USA). Each X-ray holder was individualized with an occlusal record to standardize the procedure. Radiographs were taken immediately after implant placement, at time of cementation of the final crown, and at 1, and 3 and 5 year's follow-up intervals. All radiographs were processed according to time/temperature guidelines (bath at 20°C for 4 min), digitalized (Kodak Eos camera equipped with 1/1–100 mm macro lens), and stored in JPEG format. Measurements were performed with the aid of a digital image processing software (DBSWIN 5, DÜRR DENTAL AG, Germany) used to calculate vertical distance between bone levels and implant neck at calibrated 10× magnification (Figure 1). The same oral and maxillofacial radiologist interpreted all radiographs in order to avoid operator variations.

Marginal bone level, relative to the implant reference point (implant shoulder), was measured twice to the nearest 0.1 mm mesial and distal to the implants at four time intervals: implant placement (T1), after the cementation of final crown at 8 weeks (T2), 1 year (T3), 3 years (T4), and 5 years (T5) of functional loading. The mean value of these two measurements was calculated for each implant.

### Statistical analysis

The primary outcome variable was the change of marginal bone level from baseline to the follow-up examinations at 8 weeks, 1 year, 3 years, and 5 years after loading.

The linear mixed model analysis was used to detect significant changes in marginal bone levels with time. This analysis accounts for the inherent correlation between repeated measurements on the same patient. At each time point, MBL at the mesial (M) and distal (D) surfaces were compared using the paired Student *t*-test.

A value of *p* < 0.05 was considered statistically significant. Analyses were carried out using STATA software version 10.0 (Stata Corp LP, College Station, TX, USA) and SPSS software version 18.0 (SPSS Inc., Chicago, IL, USA).

## Results

Inter-examiner correlation revealed non-significant error margin (*P* < 0.01). Statistical analysis revealed that MBL was more pronounced in implants inserted in fresh extraction sockets (*P* < 0.041) compared to healed sockets (*P* < 0.54). Radiological findings are summarized in Table 1.

**Table 1:** **Mean and *SD* of MBL (in mm) at different time periods on an implant level**.

	**T**	**Mesial**	**Distal**	**Average**
Immediate	T2	0.065 ± 0.143	0.213 ± 0.270	0.139 ± 0.165
	T3	0.278 ± 0.239	0.257 ± 0.221	0.267 ± 0.161
	T4	0.240 ± 0.214	0.290 ± 0.247	0.265 ± 0.171
	T5	0.208 ± 0.247	0.217 ± 0.252	0.213 ± 0.185
Healed	T2	0.169 ± 0.221	0.319 ± 0.246	0.244 ± 0.17
	T3	0.181 ± 0.231	0.350 ± 0.203	0.266 ± 0.176
	T4	0.138 ± 0.202	0.3 ± 0.22	0.219 ± 0.175
	T5	0.125 ± 0.175	0.263 ± 0.22	0.194 ± 0.172

When the linear mixed model was fitted, it was found that time had a significant effect on the average MBL (*P* = 0.005) and mesial MBL (*P* = 0.006), but not on distal MBL (*P* = 0.213). The outcome of the test is displayed in Table 2.

**Table 2:** **Statistical outcome of MBL using the linear mixed model**.

	**Average *P***	**Mesial (mm)**	**Distal (mm)**
T2	0.527	0.085	0.88
T3	0.007	0.014	0.172
T4	0.034	0.145	0.077
T5	0.079	0.183	0.097

Using the paired *t*-test, we found that there was a statistically significant difference between the mesial and distal measurements at T1 for all the samples (*P* = 0.003) but not afterwards as shown in Table 3.

**Table 3:** **MBL difference between mesial and distal measurements at different time points (in mm)**.

	**T2**	**T2**	**T3**	**T3**	**T4**	**T4**	**T5**	**T5**
	**Mean ± *SD***	***P***	**Mean ± *SD***	***P***	**Mean ± *SD***	***P***	**Mean ± *SD***	***P***
IMME temp	0.148 ± 0.28	0.019	0.022 ± 0.33	0.754	0.05 ± 0.31	0.480	0.08 ± 0.34	0.933
Healed temp	0.150 ± 0.32	0.081	0.169 ± 0.26	0.019	0.162 ± 0.24	0.030	0.138 ± 0.20	0.092

## Discussion

The results of this study showed that that there was no significant difference in bone loss between the two investigated groups. Careful analysis of data revealed that the majority of MBL was observed during the first year of loading after which the rate bone loss remained relatively constant: 0.01–0.02 mm/year. Interestingly, some bone loss was regained after a period of 5 years. MBL values reported in this study were lower compared to other studies with similar observation periods (Boronat et al., 2008; Collaert and De Bruyn, 2008; Testori et al., 2008; Tözüm et al., 2008; Bergkvist et al., 2009; Piao et al., 2009; Pikner and Gröndahl, 2009; Pikner et al., 2009; Song et al., 2009).

**Figure 2:**
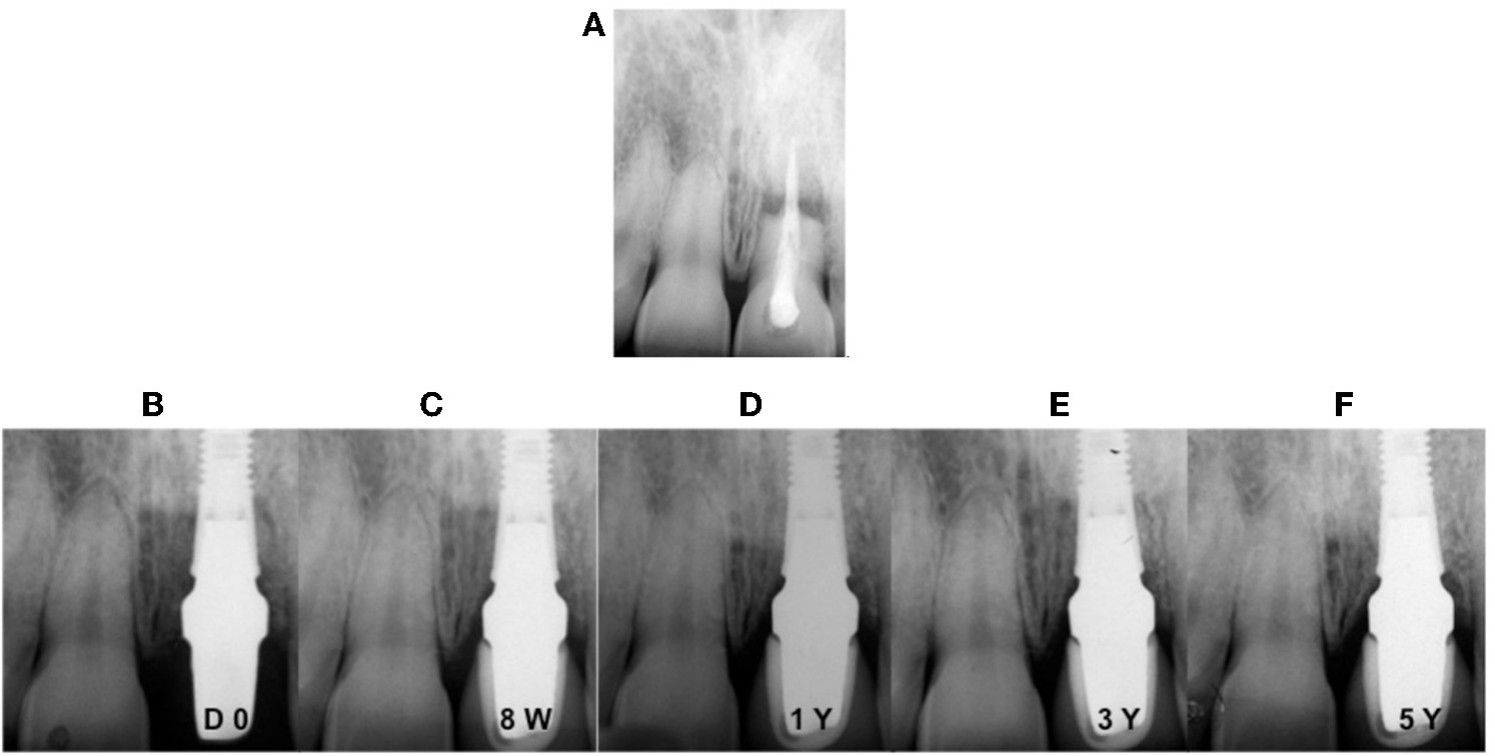
**Digital intra-oral radiographs of implants placed in fresh extraction socket with immediate loading at different observation times. (A)** Pre-operative situation; **(B)** placement of the abutment and temporary crown; **(C)** 8 weeks after definitive crown cementation; **(D)** after 1 year; **(E)** after 3 years; **(F)** after 5 years.

**Figure 3:**
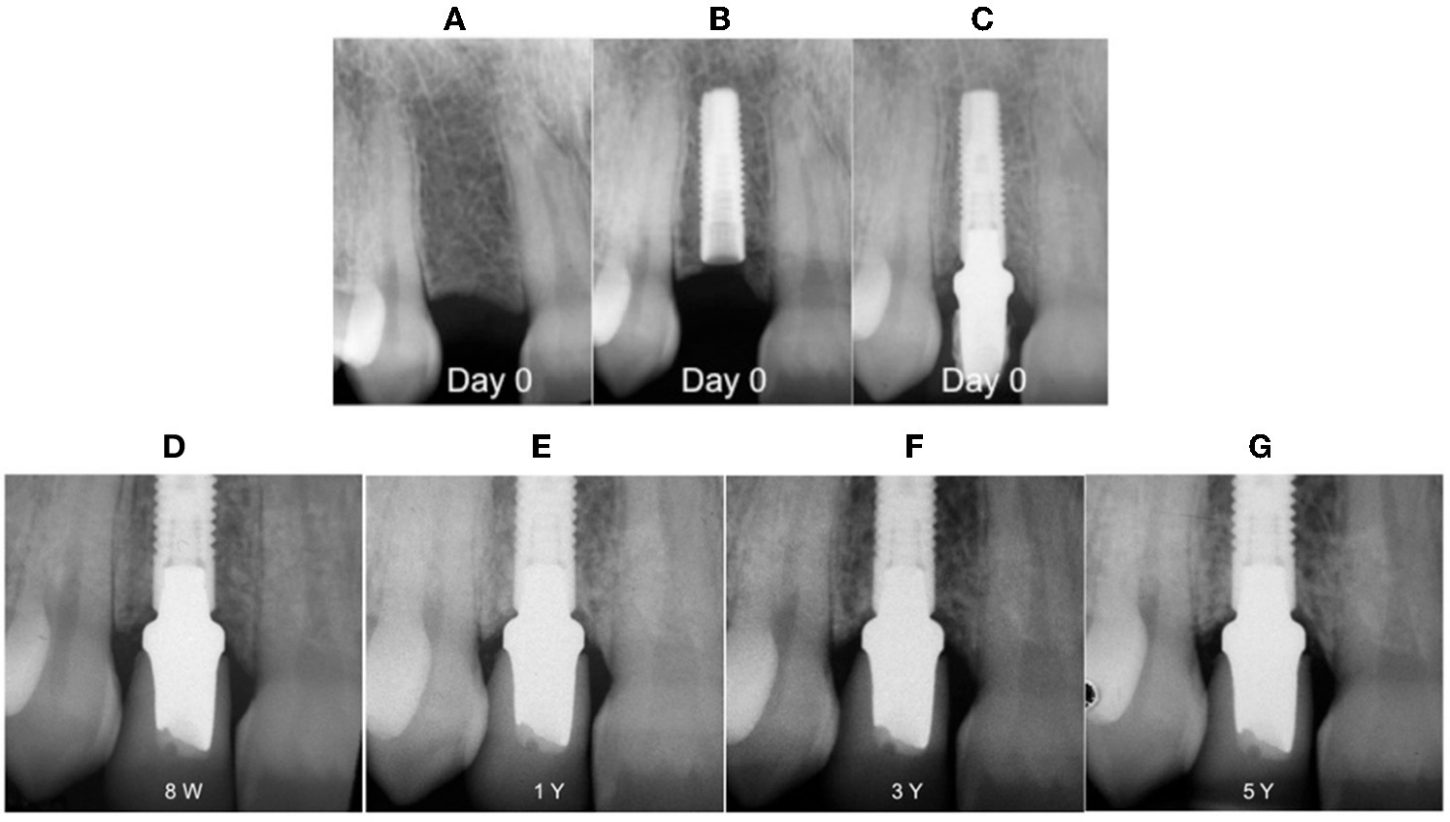
**Digital intra-oral radiographs of implants placed in healed ridge. (A)** Pre-operative situation; **(B)** implant placement; **(C)** abutment placement; **(D)** 8 weeks after definitive crown cementation; **(E)** after 1 year; **(F)** after 3 years; **(G)** after 5 years.

Bone loss for healed sites (group I) in our study was about 0.266 ± 0.176 mm while with another study it showed an increase of up to 0.78 mm (Ericsson et al., 2000), which can be explained by the formation of the biological width (Hermann et al., 2001). In another reports, with the same implant systems, the mean value of MBL was 0.40 ± 1.43 mm (Cooper et al., 2010) and 0.40 ± 1.51 mm (De Bruyn et al., 2013) for a 3 years observation period. On the other hand, marginal bone level changes showed a gain of 0.02 mm at 5 years for the delay group and 0.05 for the immediate group (group II), which was greater than what was found in other studies (Kan et al., 2003; De Rouck et al., 2008, 2009). Clear differences between healed sites (Figure 2) and extraction sockets (Figure 3) are associated with the bone fill occurring in the space between implants and post-extraction sockets during osseous healing (Paolantonio et al., 2001; Araujo et al., 2006).This accounts for the apparent gain or minimal change in marginal bone levels over the observation period (Kan et al., 2003; Norton, 2004; De Rouck et al., 2009).

Interproximal loss of marginal bone levels of 0.266 mm (*SD* = 0.26) after 12 months is in agreement with other reports on the use of the Astra Tech Implant System in healed ridges. It is consistent with those obtained in an early loading study (Cooper et al., 2007, 2010). Recently, Donati et al. (2013) demonstrated mean changes in marginal bone levels of 0.17 mm (*SD* = 0.66) for 4.0-mm implants and 0.48 mm (*SD* = 1.0) for 4.5-mm implants at 1 year following immediate functional loading. Whereas other investigations compared bone level changes at immediately loaded vs. conventionally loaded implants (Lindeboom et al., 2006; Crespi et al., 2008) or immediate loading vs. immediate provisionalization (Nisapakultorn et al., 2010) and did not identify differences in interproximal marginal bone levels. This study recorded similar resultant interproximal bone-to- implant contact levels after the immediate provisional loading of implants placed in healed ridges (group I) vs. extraction sockets (group II). Higher MBL was observed on mesial surfaces compared to distal surfaces of the implants. This observation could be related to the anatomical features such as incisive fissure or interdental septum, or due to the direction of stress distribution around the neck of the implant (Woelfel and Scheid, 2002; Palmer et al., 2007). After 5 years, the difference in marginal bone levels between mesial and distal sites requires periodic evaluation in order to maintain acceptable levels of oral hygiene. The reason for the apparent lower rate of MBL may be due to the association of implant insertion with final abutment connection without any later manipulation. These findings are in accordance with previous studies on the effect of abutment dis/reconnections on peri-implant bone resorption (Carlson et al., 2004; Canullo and Rasperini, 2007; Testori et al., 2008; Bergkvist et al., 2009; Canullo et al., 2010). Berglundh et al. (2005) analyzed marginal bone alterations following implant placement, abutment connection, and functional loading; they reported that the largest amount of bone loss occurred following implant placement and abutment connection and that almost no bone level alterations occurred after. These findings are in accordance with our results and other clinical reports (Botticelli et al., 2008; Nisapakultorn et al., 2010; Valentini et al., 2010). The results of the present study indicate that insertion of immediately loaded implants in fresh extraction sockets (group II) result in significant reduction of resorption of marginal ridge.

Under the guidelines for treatment established by the inclusion and exclusion criteria, immediate loading is a safe and efficacious procedure when measured in terms of implants survival. This study has limitations due to the number of cases and implants. Also some limitations are related to the immediate placement in fresh extraction sockets, it's presently advocated that the depth of implant placement be no less than 2–3 mm apical to the adjacent clinical crown margin. Further, it is recommended that the implant abutment interface should not be placed beyond the facial crest.

## Conclusion

Within the limitations of this study, and the small number of cases, the association between implant placement and final abutment connection in the immediate loading protocol seems to reduce MBL and soft tissue collapse. Immediate loading in fresh extraction of healed sockets was not associated with increased MBL.

### Conflict of interest statement

The authors declare that the research was conducted in the absence of any commercial or financial relationships that could be construed as a potential conflict of interest.
